# The ADVanced Organ Support (ADVOS) Hemodialysis System in Postoperative Cardiogenic Shock and Multiple Organ Failure: First Results in Cardiac Surgery Patients

**DOI:** 10.3390/life15071042

**Published:** 2025-06-30

**Authors:** Veronika Walter, Ekaterina Hinrichs, Tarek Alloush, Aritz Perez Ruiz de Garibay, Gregor Warnecke, Wiebke Sommer, Hanna Gravert, Christina Grothusen, Janine Becker, Alexander Thiem, Bernd Panholzer

**Affiliations:** 1 Department of Cardiac Surgery, University Hospital Schleswig-Holstein, Campus Kiel, 24105 Kiel, Germany; veronika.walter@uksh.de (V.W.); ekaterina.hinrichs@uksh.de (E.H.); mohamadtarek.alloush@uksh.de (T.A.); gregor.warnecke@uksh.de (G.W.); wiebke.sommer@uksh.de (W.S.); hanna.gravert@uksh.de (H.G.); christina.grothusen@uksh.de (C.G.); janine.becker@uksh.de (J.B.); alexander.thiem@uksh.de (A.T.); 2 ADVITOS GmbH, 80992 Munich, Germany; aritz.perez@advitos.com

**Keywords:** cardiac surgery, liver failure, cardiogenic shock

## Abstract

Background: The management of multiple organ failure in the vulnerable cohort of cardiac surgery patients with cardiogenic shock remains a significant challenge, often impairing patient survival. A multimodal approach at targeting organ dysfunction seems to be a promising strategy, encompassing both hemodynamic support as well as differentiated organ replacement therapy. Materials and Methods: In our retrospective study we examined the impact of the ADVOS (advanced organ support) system on overall outcomes and survival in an all-comers group of 22 cardiac surgery patients with postoperative cardiogenic shock and multiple organ failure. Aims: The objective of the study was to assess the feasibility and potential benefits of ADVOS treatment in this patient population. Results: The standard care management in combination with ADVOS therapy corrected acid–base balance (pH 7.33 vs. 7.44, *p* = 0.001; base excess −3.2 vs. 2.4 mmol/L, *p* < 0.001). This contributed to restoring hemodynamic balance after two consecutive ADVOS treatments (vasoactive inotropic score (VIS) 59 vs. 21, *p* = 0.007, noradrenaline 0.470 vs. 0.180 µg/kg/min, *p* = 0.009). Conclusions: Our findings indicate that ADVOS treatment is both feasible and safe, with a substantial proportion of patients demonstrating improvements in organ function and overall outcomes.

## 1. Introduction

Cardiac surgery patients may face significant challenges during the postoperative period, one of the most severe being cardiogenic shock (CS). Cardiogenic shock is defined as a state of peripheral hypoperfusion due to reduced cardiac output, typically resulting from myocardial infarction or severe heart failure [1]. This condition frequently leads to multiple organ failure (MOF), characterized by the simultaneous dysfunction of two or more organ systems, which poses a substantial risk to patient survival [2,3].

The importance of addressing CS in postoperative cardiac surgery patients cannot be overstated. Despite advancements in surgical techniques and postoperative care, CS remains a leading cause of morbidity and mortality in this population [4]. Effective management strategies are crucial to improving survival rates and quality of life for these patients. The high mortality associated with CS and its complications underscores the need for innovative therapeutic approaches that can provide comprehensive organ support during the critical postoperative period [5,6].

Current treatment options for CS include pharmacological interventions, mechanical circulatory support, and organ-specific therapies [7]. However, these treatments often fall short in managing the complex pathophysiology associated with MOF [8,9].

ADVOS (advanced organ support) therapy is an emerging extracorporeal treatment designed to provide multi-organ support by simultaneously assisting liver, kidney, and lung functions. The ADVOS multi-hemodialysis system (ADVITOS GmbH, Munich, Germany) combines renal and hepatic support (through albumin dialysis), lung support (through fluid-based CO_2_ removal), fluid management and acidosis correction, aiming to stabilize hemodynamics and enhance organ recovery [10,11,12,13]. Initial studies have shown promise in the use of ADVOS for patients with liver and kidney failure, but its feasibility and effectiveness in the context of postoperative CS have not been explored. This gap highlights the need for further research to evaluate the potential benefits and limitations of ADVOS therapy in a real-world, all-comers situation.

This study aims to address this gap by investigating the feasibility of ADVOS therapy in a diverse cohort of cardiac surgery patients who develop postoperative CS and MOF. The research questions guiding this study include the following: (1) Is ADVOS therapy safe and feasible in the management of postoperative cardiogenic shock and MOF? (2) What are the clinical outcomes associated with the use of ADVOS in this patient population? (3) How does ADVOS therapy impact hemodynamic stability and overall survival rates?

## 2. Materials and Methods

### 2.1. Patients and Study Design

We conducted a retrospective observational study at the cardiac surgery intensive care unit of the Clinic for Cardiovascular Surgery from the University Hospital Schleswig-Holstein in Germany. All patients treated with advanced organ support (ADVOS, ADVITOS, GmbH, Munich, Germany) for at least two consecutive sessions from November 2021 to September 2023 were included in the analysis. All of the included patients suffered from multiple organ failure and refractory post-cardiotomy cardiogenic shock (PCCS), requiring inotropes and/or vasopressors. To reduce coincidental effects of PCCS/MOF and timing of therapy escalation, patients with only one cycle of ADVOS treatment were excluded from the analysis.

The primary objective was the reduction in vasoactive inotropic score (VIS) after 48 h. The course of hemodynamic, hepatic, renal, ventilation and blood gas parameters as well as mortality rates at 28 days and ICU discharge were documented.

The study followed the principles of the Declaration of Helsinki (as revised in 2013). and was approved by the Ethics Board of the Christian Albrecht University of Kiel, Germany (No. D 428/23). Due to the retrospective nature of the study, individual consent was waived.

### 2.2. ADVOS Hemodialysis System

The ADVOS multi-hemodialysis system (ADVITOS GmbH, Munich, Germany) was used according to its intended purpose, as described in the instructions for use of the manufacturer: remove water-soluble toxic substances and protein-bound toxic substances, normalize or improve the composition of blood in case of, e.g., electrolyte or acid–base disturbances (e.g., metabolic acidosis or respiratory acidosis) and remove fluids in case of fluid overload.

**Figure 1:**
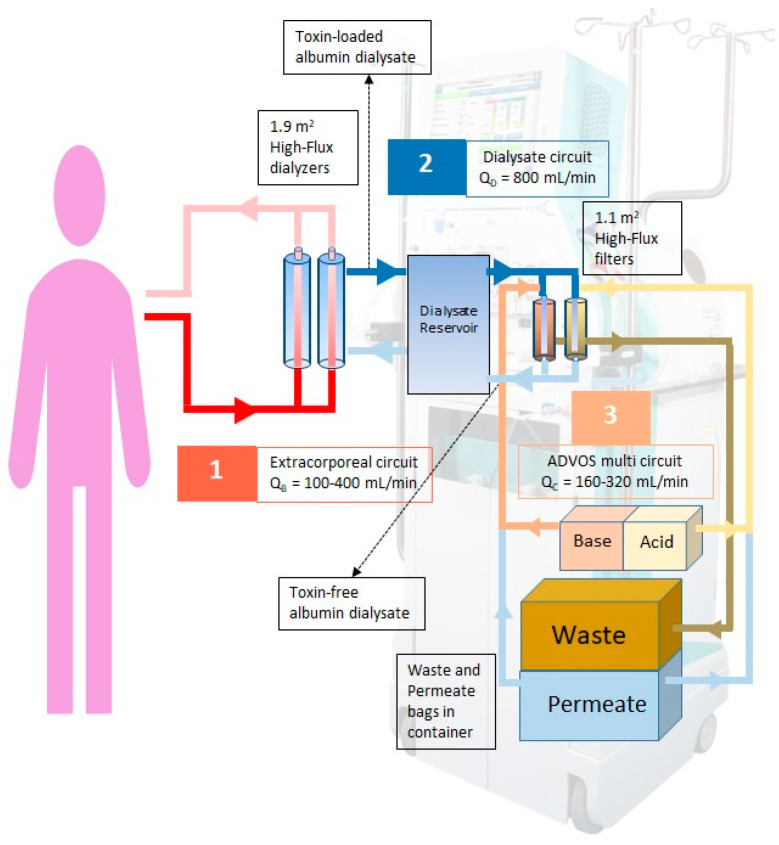
ADVOS multi hemodialysis system’s schematic representation. 1: Extracorporeal circuit: patient blood; 2: Dialysate circuit: albumin enriched dialysate; 3: ADVOS multi circuit: Through alkalization and acidification, the toxins are removed from the albumin so that it can be reused.

To exert these effects, the ADVOS multi-device consists of three circuits (Figure 1). Blood is circulated (100–400 mL/min) through the extracorporeal circuit containing two ELISIO 19H dialyzers (Nipro D.Med Germany GmbH, Hamburg, Germany). The dialysate, enriched with human albumin (200 mL, 20%), flows co-currently from the dialysate circuit (800 mL/min) and facilitates the transfer of toxins from the blood. The toxin-laden dialysate then moves into the ADVOS multi-regeneration circuit, where alterations in pH and temperature induce structural changes in the dialysate albumin, allowing it to release protein-bound toxins. These toxins, along with water-soluble toxins, are subsequently removed by convection through two ELISIO 11H filters (Nipro D.Med Germany GmbH, Hamburg, Germany) into the waste stream. The volume of dialysate removed is replenished with fresh dialysate (i.e., concentrate flow of 160–320 mL/min), produced by the continuous online mixing of osmosis water with an alkaline concentrate (primarily NaOH) and an acidic concentrate (primarily HCl). The final pH of the dialysate (7.2–9.5) can be individualized during the treatment and is determined by the ratio of acidic to alkaline concentrate.

### 2.3. Intervention

**Figure 2:**
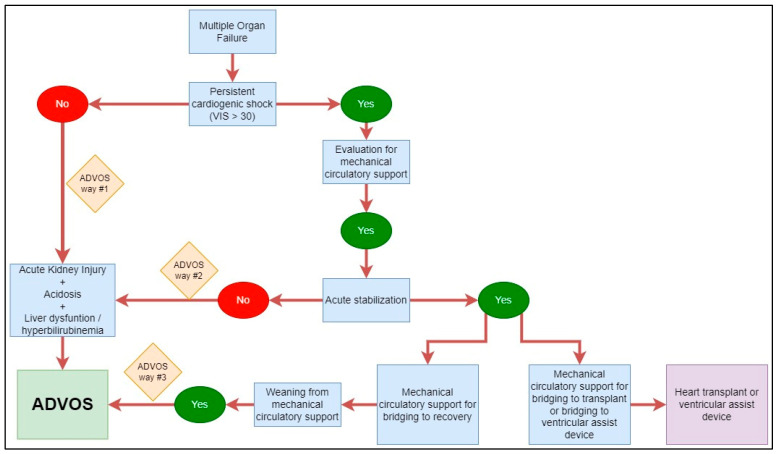
Decision tree to decide whether ADVOS therapy is appropriate for multiple organ failure patients in our cardiac intensive care unit.

Patients received a standard of care for refractory cardiogenic shock according to internal guidelines. The ADVOS hemodialysis system was used following the scheme in Figure 2. Patients with kidney failure, hyperbilirubinemia and acidosis (arterial blood pH < 7.35) that either had a VIS ≤ 30 (i.e., ADVOS way #1) or were hemodynamically unstable despite mechanical circulatory support (MCS) (i.e., ADVOS way #2) were eligible for early ADVOS. In patients where MCS allowed acute hemodynamical stabilization and bridging to recovery (BTR), ADVOS was set to facilitate the weaning process after explantation of the MCS system (i.e., ADVOS way #3).

### 2.4. Data Collection and Analysis

Routine laboratory parameters such as bilirubin, creatinine, blood urea nitrogen (BUN), procalcitonin, electrolyte levels, IL-6, lactate, erythrocytes, leukocytes, platelets and blood gas analysis were recorded. Ventilation and mechanical circulatory parameters as well as ADVOS settings were also assessed. Additionally, clinical data encompassing pre-existing conditions and catecholamine dosages were documented. For analysis, the parameters immediately before and after each ADVOS session were examined. Finally, the vasoactive inotropic score and the standardized mortality rates were calculated.

#### 2.4.1. Vasoactive Inotropic Score (VIS)

The vasoactive inotropic score (VIS) is a quantifiable measure used to assess the cumulative dosage of vasoactive and inotropic medications administered to a patient [14]. It provides an objective means to gauge the level of cardiovascular support required. The VIS is calculated using the following formula:

VIS = dopamine dose (μg/kg/min) + dobutamine dose (μg/kg/min) + 100 × epinephrine dose (μg/kg/min) + 100 × norepinephrine dose (μg/kg/min) + 10 × milrinone dose (μg/kg/min) + 10,000 × vasopressin dose (IU/kg/min)

#### 2.4.2. Standardized Mortality Ratio (SMR)

The standardized mortality ratio (SMR) compares the mortality rates of a known reference population with a specific population to assess the relative risk of death. The SMR was determined by comparing the actual number of deaths among treated patients to the expected number based on their highest baseline SOFA scores, assessed before the initial ADVOS session. Patients were grouped by expected mortality rates as outlined by Ferreira et al. [15]. The expected deaths for each group were calculated and aggregated to obtain the total expected deaths:

SMR = expected deaths/observed deaths

The absolute risk reduction (ARR) and number needed to treat (NNT) were also calculated from the expected and observed death data.

ARR = expected deaths (%) − observed deaths (%)

NNT = 1/ARR

### 2.5. Statistical Analysis

Unless specified otherwise, variables were presented as median values with the interquartile range (IQR) spanning the 25th to 75th percentiles. Levene and Shapiro–Wilk tests were performed to assess the homogeneity of variance and the normal distribution of data, respectively. Paired sample comparisons before and after two consecutive ADVOS sessions were conducted using Student’s t-test for dependent variables. A two-tailed *p*-value of less than 0.05 was considered statistically significant. Data analysis was performed using IBM SPSS Statistics for Windows, version 28.0.

## 3. Results

### 3.1. Baseline Characteristics

Study patients were mostly male (77%) with a median of 59 years (IQR 51, 65). All except for one required vasoactive and supportive medication and were mechanically ventilated (95%) at the time of starting ADVOS. Multiple organ failure was present in every patient with a median SOFA score of 15 (IQR 14, 18). Additionally, 95% had secondary acquired liver injury/failure. Even though the patients of our cohort presented with acute rather than acute-on-chronic liver failure and had no previous diagnosis of chronic liver disease, the course of the organ damage mimicked that of acute-on-chronic liver failure. Symptoms like ascites, secondary sclerosing cholangitis and failure of liver protein synthesis were present with an early onset.

To assess the severity of liver failure and provide comparability, the CLIF-C (Chronic Liver Failure Consortium) score was applied.

Acidosis was present in 64% of the participants. Table 1 shows these data in detail. Associated comorbidities as well as prior surgical procedures are depicted in Table 2.

### 3.2. Extracorporeal Treatment Settings

ADVOS was used in 110 sessions with a median duration of 23 h and a median number of four treatments per patient. The number of treatments per patient was higher when ADVOS was used for ECLS weaning (6 vs. 3). The blood flow was predominantly set to 150 mL/min. Citrate anticoagulation was employed in all but four sessions, and heparin was additionally required in 75% of the sessions. Only four ADVOS sessions had to be stopped earlier than planned. The main settings of ADVOS, ECLS and Impella are shown in Table 3.

### 3.3. Performance and Outcome

#### 3.3.1. Vasoactive Inotropic Score

**Figure 3:**
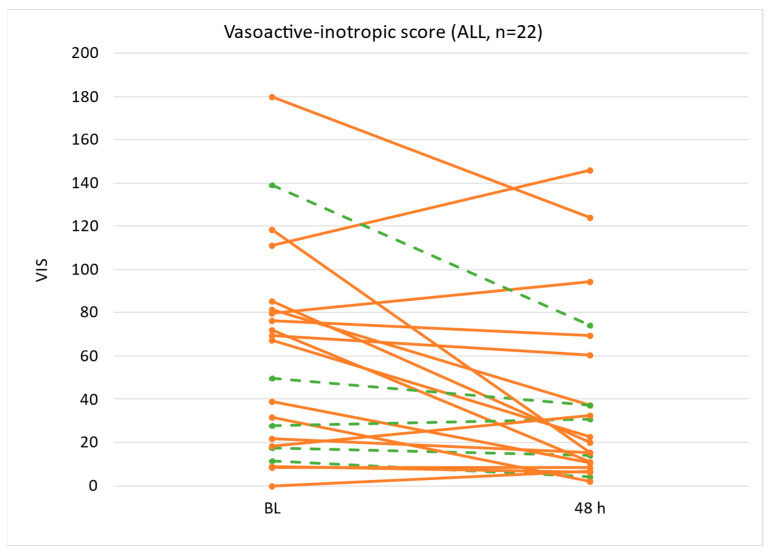
Vasoactive inotropic score at baseline (BL) and after two consecutive ADVOS treatments (48 h). The dashed green lines represent patients that received ADVOS for ECLS weaning, and orange lines represent other purposes.

The VIS decreased in 73% of the patients after two consecutive 24 h ADVOS sessions (Figure 3, Table 4). The median baseline VIS value improved significantly (*p* = 0.007) from 59 (IQR 19, 81) to 21 (IQR 11, 55). The median VIS reduction was 21 points. The dose of noradrenaline needed for a sufficient mean blood pressure decreased accordingly (*p* = 0.009) from 0.470 µg/kg/min (IQR 0.125, 0.755) to 0.180 (IQR 0.071, 0.435).

#### 3.3.2. Change of Treatment Performance Parameters After Two Consecutive ADVOS Sessions

Laboratory values and paired differences from the main performance parameters during the first 48 h of ADVOS therapy are shown in Table 5. A significant reduction in creatinine and BUN could be observed. Moreover, blood gas levels including blood pH, serum bicarbonate and base excess were corrected. Electrolyte levels were maintained in physiological ranges, and no significant changes were observed in blood count. Although not statistically significant, a trend towards lactate and IL-6 reduction was documented.

#### 3.3.3. Standardized Mortality Ratio

**Figure 4:**
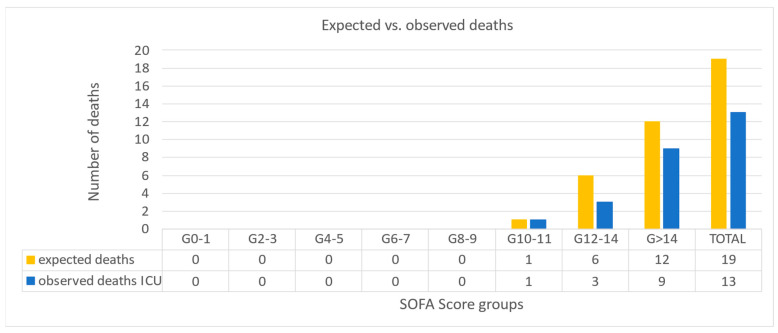
Expected deaths according to SOFA score obtained before the first ADVOS session (yellow) vs. observed deaths during ICU stay (blue) among different subgroups of SOFA score based on the publication from Ferreira et al. [15].

Twenty-one out of twenty-two patients had a documented SOFA score before the first ADVOS session. The median SOFA score in these patients was 15 (IQR 14, 18) (Table 1). As shown in Figure 4, a total of 19 deaths (90.5%) were expected according to the SOFA score. The observed mortality rate in the ICU was 61.9% (13/21 patients). As a result, the calculated SMR was 0.68 (CI 95%: 0.31–1.06). The absolute risk reduction was 29%, with a number needed to treat of 3.5. The observed ICU mortality rate was below the expected rate in both the subgroup for ECLS weaning (20%) and for other purposes (75%).

## 4. Discussion

The application of the ADVOS multi-hemodialysis system in our cohort of cardiac surgery patients with postoperative cardiogenic shock and multiple organ failure proved to be feasible and safe. The standard care management in combination with the ADVOS therapy reduced renal dysfunction parameters and corrected the acid–base balance. This contributed to restoring hemodynamic balance, as observed by a significant reduction in vasopressor and inotropic medication after two consecutive ADVOS treatments. Finally, these benefits could be translated into an improvement in expected ICU survival according to their SOFA score obtained before the first ADVOS session.

### 4.1. Interpretation and Generalizability

The management of postoperative cardiogenic shock in the cardiac ICU is challenging. It requires early recognition with an identification of the underlying cause [5]. Patients might need initial oxygen supplementation (including mechanical ventilation) and hemodynamic stabilization through fluid administration, as well as vasopressor and inotropic medication [6,16,17]. At some point, vasopressor requirements exceed recommendations, and temporary mechanical circulatory support is needed to provide additional hemodynamic stability [18,19]. Although the stance on MCS utilization for acute cardiogenic shock caused by myocardial infarction remains the subject of controversial observations regarding outcome [20,21], both the guidelines of the European Society of Cardiology (ESC) those of the International Society for Heart and Lung Transplantation/Heart Failure Society of America (ISHLT/HFSA) recommend early MCS implementation in cardiogenic shock, with recommendation class IIA and IB, respectively [22,23]. While the ECLS shock trial claims that no benefit in outcome can be derived from early MCS treatment in acute cardiogenic shock [20], the findings of the DANGER shock trial conclude that even in the presence of possible complications of percutaneous MCS implantation, there is a survival benefit for these very critical patients [21]. Further consideration of all aspects of cardiogenic shock treatment remains to be re-evaluated, especially since both trials did not cover post-cardiotomy cardiogenic shock patients. There are already several centers that conducted single-center observations with favorable outcomes due to MCS use [24]. To provide both standardized as well as adequate treatment within the concept of an early goal directed therapy, the treating center or unit needs a comprehensive MCS start protocol to enable a facilitation of decision making for the team of health care providers/physicians. The main goal of MCS in cardiogenic shock can be summarized as an increase in oxygen supply through restoration of microcirculation in affected organs [25,26]. Even under stabilizing treatment, cardiogenic shock can be complicated by multiple organ failure that would involve the use of organ-specific extracorporeal support [6,27,28,29]. Indeed, pre-emptive renal replacement therapy has been postulated as an effective approach in PCCS patients [30].

Considering this, our group defined a specific protocol (“The Kiel approach”) for the management of multiple organ failure on top of postoperative CS, which included the use of the ADVOS multi-hemodialysis system (Figure 2). This protocol defined three ways for a patient to be elective for ADVOS therapy: (1) patients without persisting CS (VIS < 30) but with acidosis and renal and/or liver dysfunction; (2) patients with persistent CS that needed mechanical circulatory support but were not able to reach acute stabilization; or (3) patients who could be stabilized with MCS but received ADVOS to facilitate the weaning process. In all these patients, the improvement in renal parameters and the correction of acidosis provided by ADVOS positively impacted the need for vasopressors, reducing the VIS score and directly facilitating the restoration of hemodynamic stability.

Systemic acidosis can occur due to hypoperfusion and concomitant kidney injury, further affecting the acid–base balance [31]. Likewise, lung dysfunction may limit compensation due to the inability to properly eliminate CO_2_ loads. The presence of acidosis is associated with harmful physiological effects in critically ill patients, including inflammatory processes [32], disruption of immune functions [33], alteration of hemoglobin’s oxygen affinity [34], induction of pulmonary vasoconstriction [35], decrease in glomerular filtration rate [36], reduction in intestinal motility [37], impairment of coagulation [38] and dysfunction of myocardial contractility [39]. In fact, Jentzer et al. demonstrated that low blood pH was independently associated with mortality in patients with CS [40].

The ADVOS multi-hemodialysis system can correct acidosis by mimicking natural physiological processes [41]. The dialysate within the ADVOS device can be individualized in terms of pH and bicarbonate content during the treatment. This allows the removal of excess acid and equilibrate bicarbonate levels through a concentration gradient as performed by the kidney [42,43]. Thus, both metabolic or respiratory acidosis could be managed by simply increasing or reducing the bicarbonate content of the dialysate, respectively, while setting a high dialysate pH [41]. This occurred at a median low blood flow of 150 mL/min. Allescher et al. already showed a rapid acid–base correction and a median CO_2_ removal of 49 mL/min with ADVOS in a cohort of COVID-19 patients [11]. Moreover, Fuhrmann et al. suggested that the improvement of acid–base balance could exert an effect on hemodynamic stability by reducing noradrenaline needs in critically ill patients treated with ADVOS for MOF [12].

The correction of acid–base balance is important not only from a physiological perspective but also from a pharmacological viewpoint. In this regard, Bauer et al. reported a diminished hemodynamic response to vasopressin due to low arterial pH in a large cohort of patients with MOF and septic shock [44]. A drop in arterial pH impairs the vasoactive effect of both naturally occurring and administered catecholamines [45,46]. Simultaneously, acidosis triggers the endogenous release of vasopressin, depleting the pituitary vasopressin reserve and further promoting vasodilation [44,47]. Bearing this in mind, we hypothesize that the rapid restoration of the acid–base balance with ADVOS allowed a reduction in vasopressor needs and a lower VIS in our study (Figure 3).

**Figure 5:**
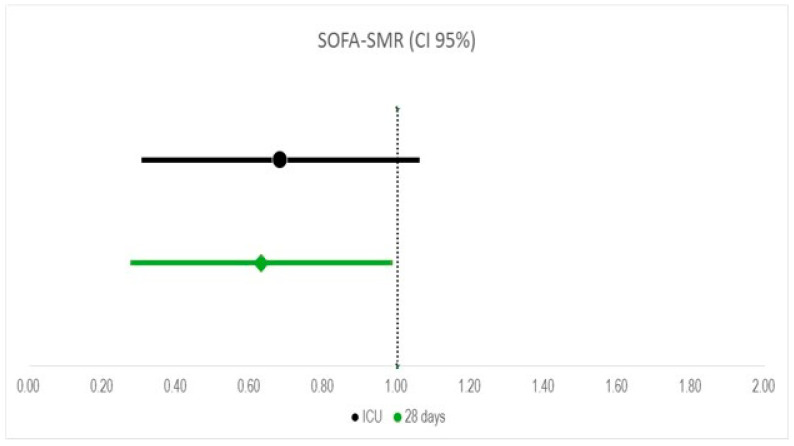
SOFA score standardized mortality ratio (SOFA-SMR) based on 28 days (green) and ICU mortality (black). Values to the left of the dotted line indicate lower mortality ratios than expected according to the SOFA Score.

The ultimate goal of hemodynamic stabilization would be an improvement in patient survival. In the absence of a control group, we calculated the standardized mortality ratio based on the SOFA score before the start of ADVOS [15]. The SMR in our cohort was 0.68 with an absolute risk reduction of 29% in comparison to the expected high mortality rate (Figure 5). The case of the subgroup of patients treated with ADVOS to facilitate ECLS weaning was of particular interest, as four out of five patients survived the ICU stay despite a median SOFA score of 16. Nonetheless, more data are needed to conclude whether ADVOS can contribute to reducing the “ECMO-gap” [48], which results in a mortality rate of 25% after successful ECLS explantation in the first 24 h and up to 50% before hospital discharge [8]. Recent studies try to elaborate mortality prediction by applying the SCAI stratification on cardiac surgery patients [49,50]. Though designed for medical cardiogenic shock patients, the SCAI shock classification is widely applicable for surgical patients with comparable parameters. Mortality prediction scores are subject to both selection and interpretation bias, so applying these scores to our patient group helped set the hypothesis for mortality reduction. The limited applicability further emphasizes the need for a higher-powered study to underline the postulated hypothesis.

### 4.2. Limitations

The results of our study have several limitations. First, only patients with at least two consecutive ADVOS treatments were included, which implies some selection bias. Our patients were critically ill with a median SOFA score of 15 and very few chances of surviving. Some of them thus died during the first treatment. The cohort of patients with only one treatment added up to five patients throughout the course of our observation. Some of them received both MCS support as well as ADVOS treatment as a bailout therapy, with an accordingly high SOFA score and SCAI classification. The defining features of the patient group with only one ADVOS treatment were hemodynamic instability despite temporary or permanent mechanical circulatory support, and refractory lactate acidosis as a result of cardiogenic shock, indicating advanced multiple organ failure. In at least two of the excluded patients, the situation was aggravated by a combined shock situation with ongoing septic shock despite antibiotic treatment. Therefore, we considered that to be able to assign some sort of effect to the ADVOS therapy, at least 48 h of observation should be required. Second, due to the low number and the heterogenicity of the patients, the generalizability of the results is limited. However, the improvement in some performance parameters allowed significant statistical differences to be observed. While the small number of patients does not permit a statistic interpretation applicable to a potential larger group, the observed changes in parameters could guide the design of larger studies in the future, with a focus on varied subgroups in larger numbers. This could guide the design of larger studies in the future. Finally, the absence of a control group makes it difficult to draw conclusions about the improvement in expected mortality rates. The aim of this study was to establish a concept in the complex treatment of critically ill patients, with employment of all treatment methods available at our center. Given the life-threatening nature of the state these patients found themselves in, a propensity-matched control group with the same conditions was not available at the time of the observation. All patients received the same kind of therapy escalation. Nevertheless, previous reports with ADVOS point in the same direction [12,13,51,52,53], suggesting that randomized controlled trials should be carried out in a timely manner to confirm these results.

Data integrity was ensured by assigning a team of physicians and a study nurse not affiliated with the company to collect and curate the datasets. ADVITOS GMBH was not involved in the conceptualization of the study, nor was any funding provided. The use of the ADVOS system was applied according to the standard of care on our ICU.

## 5. Conclusions

Our results demonstrate the feasibility of ADVOS therapy in patients with post-cardiotomy cardiogenic shock and multiple organ failure. The ADVOS therapy helped to restore hemodynamic stability by significantly reducing the vasoactive inotropic score after two consecutive treatments. This translated into improved survival compared to that expected according to the SOFA score obtained before the start of ADVOS treatment. We hypothesize that this effect can be attributed to the effective correction of acid–base balance, which would have contributed to increasing the effectiveness of the standard management measures for cardiogenic shock.

## Figures and Tables

**Table 1:** Baseline characteristics immediately before the first ADVOS session. Median (IQR). ECLS: extracorporeal life support; BIPAP: bilevel positive airway pressure; ASB: assisted spontaneous breathing; NIV CPAP: non-invasive ventilation continuous positive airway pressure; ACLF: acute-on-chronic liver failure. VIS: Vasoactive Inotropic Score; CLIF-C ACLF: Chronic Liver Failure score Acute-on-Chronic Liver Failure; MELD score: Model for End-stage Liver Disease score; SOFA score: Sequential Organ Failure Assessment score.

Parameters	ALL (*n* = 22)
Age (years)	59 (51–65)
Sex (male, %)	77%
Body height	175 (172–178)
Body weight	88 (72–100)
Vasoactive and supportive substances (%)	95%
VIS	59 (19–81)
Mechanical ventilation (%)	95%
BIPAP	59%
ASB	36%
NIV CPAP	5%
Acidosis (%)	64%
Secondary acquired liver injury/failure	95%
CLIF-C ACLF score	57 (54–59)
n.d.	5%
ACLF grade 1	0%
ACLF grade 2	27%
ACLF grade 3	68%
MELD score	27 (25–30)
Glasgow coma score	4 (4–4)
SOFA score	15 (14–18)
SOFA cardiac	4 (4–4)
SOFA respiratory	2 (1–2)
SOFA liver	2 (1–3)
SOFA kidney	3 (2–4)
SOFA coagulation	2 (1–2)
SOFA GCS	4 (4–4)

**Table 2:** Comorbidities and prior surgeries. ECLS: extracorporeal life support; AMI: acute myocardial infarction; CS: cardiogenic shock; CPR: cardiopulmonary resuscitation; MCS: mechanical circulatory support; LVAD: left ventricular assist device; RVAD: right ventricular assist device; TAH: total artificial heart; BiVAD: biventricular assist device; HTx: heart transplantation; CABG: coronary artery bypass grafting; AVR: aortic valve replacement; MVR: mitral valve repair/replacement; COPD: chronic obstructive pulmonary disease; HFrEF: heart failure with reduced ejection fraction; HFmrEF: heart failure with mid-range ejection fraction; HFpEF: heart failure with preserved ejection fraction.

Interventions	ALL (*n* = 22)
Prior emergency surgery	9%
Prior AMI (<48 h)	0%
CS prior surgery	5%
CPR prior surgery	0%
ECLS w/o surgery	5%
Durable MCS	5%
LVAD	5%
LVAD/RVAD	0%
TAH	0%
BiVAD	0%
Post HTx	5%
Impella	18%
Combination procedure prior surgery	9%
CABG	0%
AVR	18%
MVR	9%
Aortic surgery	5%
Acute Infective Endocarditis	5%
Acute Type A Aortic Dissection	0%
COPD	0%
Pulmonary Hypertension	5%
HFrEF	5%
HFmrEF	0%
HFpEF	0%
Moderately/severely reduced right ventricular function	18%
Chronic neuropathology	5%
Chronic renal insufficiency	5%
Acute kidney injury	64%
Chronic liver insufficiency	0%

**Table 3:** Treatment settings for ADVOS and ECLS. Median (IQR).

ADVOS	ALL (*n* = 22)	
Total number of treatment sessions	110	
Treatment session/patient	4 (3–4)	
Median treatment duration (h)	23 (16–24)	
Median blood flow (mL/min)	150 (150–150)	
Median concentrate flow (mL/min)	160 (160–160)	
Median dialysate pH value	7.8 (7.6–8.0)	
Maximal dialysate pH value	7.8 (7.8–8.5)	
Median ultrafiltration volume (mL)	2072 (754–3438)	
CiCa anticoagulation (% of treatments)	98%	
Additional anticoagulation in patient (e.g., heparin) (% of treatments)	75%	
Total number of treatment abortions (*n*, %)	4 (4%)	
ECLS	ALL (*n* = 22)	
Time	BL	48 h
Number of patients on ECLS	10	7
Median blood flow (L/min)	2.6 (1.8–3.9)	4.2 (3.1–4.3)
Median sweep gas flow (L/min)	4.0 (2.6–4.0)	3.0 (2.4–4.4)
Impella	ALL (*n* =22)	
Time	BL	48 h
Number of patients on Impella	8	8
Median blood flow (L/min)	3.4 (3.1–4.1)	3.1 (2.5–4.4)

**Table 4:** Vasoactive inotropic score and noradrenaline needs at baseline and after each ADVOS session. Median (IQR). BL: baseline; Diff.: mean paired difference (lower and upper limits); Sig.: significance (*p* value).

Parameter			ALL (*n* = 22)		
	BL	24 h	48 h	Diff. (BL vs. 48 h)	Sig.
Noradrenaline highest dose (µg/kg/min)	0.470 (0.125–0.755)	0.225 (0.108–0.488)	0.180 (0.071–0.435)	−0.199 (−0.056–−0.242)	0.009
Vasoactive inotropic score	59 (19–81)	31 (17–58)	21 (11–55)	−21 (−7–−36)	0.007

**Table 5:** Course of treatment performance parameters and paired differences after 2 consecutive ADVOS sessions (48 h). Median (IQR). BL: baseline; Diff.: mean paired difference (lower and upper limits); Sig.: significance (*p* value). ECLS: extracorporeal life support; BUN; blood urea nitrogen; IL-6: interleukin-6; RBCs: red blood cells; WBCs: white blood cells.

Parameter	ALL (*n* = 22)			
	BL	48 h	Diff.	Sig.
Bilirubin (mg/dL)	5.0 (1.8–9.0)	6.6 (2.4–10.1)	0.8 (2.3–−0.6)	0.241
Creatinine (mg/dL)	1.32 (0.99–2.30)	1.04 (0.70–1.48)	−0.5 (−0.2–−0.9)	0.002
BUN (mg/dL)	12.3 (7.7–16.1)	6.3 (4.7–9.6)	−6.3 (−3.2–−9.5)	0.000
IL-6 (ng/L)	213 (94–647)	136 (105–365)	−394 (92–−879)	0.105
Procalcitonin (ng/mL)	1.3 (0.6–5.6)	3.3 (1.2–6.7)	1.5 (6.1–−3.1)	0.489
Lactate (ng/mL)	2.3 (1.7–4.7)	1.8 (1.2–2.2)	−1.7 (0.2–−3.5)	0.077
Blood pH	7.33 (7.29–7.40)	7.44 (7.38–7.44)	0.08 (0.13–0.04)	0.001
Bicarbonate (mmol/L)	22.6 (20.5–24.5)	26.8 (25.5–27.8)	4.2 (6.2–2.3)	0.000
Base excess (mmol/L)	−3.2 (−5.0-1.5)	2.4 (0.6–3.1)	5.5 (7.9–3.1)	0.000
Na^+^ (mmol/L)	141 (133–145)	138 (136–140)	−1.0 (1.8–−3.7)	0.473
Cl^−^ (mmol/L)	105 (101–110)	105 (103–108)	−1.1 (1.9–−4.1)	0.462
Phosphate (mmol/L)	1.54 (1.03–2.06)	1.07 (0.84–1.53)	−0.40 (−0.10–−0.69)	0.010
RBCs (/pL)	3.0 (2.8–3.3)	2.9 (2.7–3.1)	−0.1 (0.0–−0.3)	0.067
WBCs (/nL)	10.9 (8.5–16.8)	11.9 (9.5–16.0)	−0.2 (2.8–−3.3)	0.886
Platelets (/nL)	107 (87–150)	101 (80–122)	−24 (8–−55)	0.132
FiO2	0.50 (0.45–0.69)	0.40 (0.31–0.49)	0.13 (0.05–0.20)	0.002
PEEP (mbar)	12 (11–14)	10 (10–12)	1.3 (0.3–2.4)	0.017
Tidal Volume (mL/kg)	5.6 (4.4–6.2)	5.3 (4.7–6.5)	−0.21 (−0.7–0.3)	0.388
Driving Pressure (mbar)	12 (10–15)	12 (10–16)	−0.2 (−1.8–1.4)	0.761

## Data Availability

The datasets used and/or analyzed during the current study are available from the corresponding author on reasonable request.
